# Integrative modeling of hemodynamic changes and perfusion impairment in coronary microvascular disease

**DOI:** 10.3389/fbioe.2023.1204178

**Published:** 2023-07-26

**Authors:** Monika Colombo, Palak Chaudhry, Yvonne Oberholzer, Andrew J. deMello

**Affiliations:** ^1^ Department of Chemistry and Applied Biosciences, Institute for Chemical and Bioengineering, ETH Zurich, Zürich, Switzerland; ^2^ Department of Mechanical and Production Engineering, Aarhus University, Aarhus, Denmark

**Keywords:** 3D printing, CFD analysis, WSS, fabrication, local hemodynamics, nonobstructive coronary artery disease

## Abstract

**Introduction:** Coronary microvascular disease is one of the responsible factors for cardiac perfusion impairment. Due to diagnostic and treatment challenges, this pathology (characterized by alterations to microvasculature local hemodynamics) represents a significant yet unsolved clinical problem.

**Methods:** Due to the poor understanding of the onset and progression of this disease, we propose a new and noninvasive strategy to quantify *in-vivo* hemodynamic changes occurring in the microvasculature. Specifically, we here present a conceptual workflow that combines both *in-vitro* and *in-silico* modelling for the analysis of the hemodynamic alterations in the microvasculature.

**Results:** First, we demonstrate a hybrid additive manufacturing process to fabricate circular cross-section, biocompatible fluidic networks in polytetrafluoroethylene. We then use these microfluidic devices and computational fluid dynamics to simulate different degrees of perfusion impairment.

**Discussion:** Ultimately, we show that the developed workflow defines a robust platform for the multiscale analysis of multifactorial events occurring in coronary microvascular disease.

## 1 Introduction

There is increasing recognition of patients who present symptoms and signatures of ischemic heart disease but without obstructed coronary arteries. (Bairey Merz et al., 2017). Despite presenting nonobstructive coronary artery disease (30–50%), these patients can experience major cardiovascular events, including myocardial infarction and death, with higher frequencies observed in women than in men (Sara et al., 2015). The mechanisms contributing to this pathological condition are multifaceted, with one proposed factor being coronary microvascular dysfunction or CMD (Camici and Crea, 2007). CMD is an umbrella definition for epicardial and/or microvascular endothelial and/or non-endothelial dysfunction that limits myocardial perfusion, and has been demonstrated to be an early manifestation of atherosclerosis; most common in women suffering from nonobstructive coronary artery disease (Anderson et al., 2019; Kissel and Anderson, 2020).

CMD is often indicated by a reduced coronary flow reserve, namely, an inadequate ability of the coronary circulation to respond to a physiological increase in oxygen demands with a corresponding increase in blood flow (Zeiher et al., 1989; Díez-delhoyo et al., 2015). This is quantitively defined as the ratio between coronary blood flow at maximal hyperemia and at baseline conditions. Interestingly, patients with low coronary flow reserve have an increased risk of adverse outcomes, independent of the severity of obstructive disease in epicardial arteries (Murthy et al., 2012). Coronary vessels with diameters below 400 µm are responsible for the bulk of coronary resistance and the regulation of myocardial flow, which should be maintained independent of blood pressure and epicardial stenoses (Díez-delhoyo et al., 2015). In the case of CMD, impaired microvascular resistance and fractional flow reserve can be assessed through a number of noninvasive or invasive tests, each with specific strengths and limitations (Taqueti and Di Carli, 2018; Whayne et al., 2020). That said, CMD diagnosis and treatment remains highly challenging.

Recently, it has been shown that low wall shear stress (WSS), calculated through computational fluid dynamics (CFD), is associated with endothelial dysfunction in patients with nonobstructive coronary artery disease (Kumar et al., 2018). Coronary fractional flow reserve in epicardial arteries was shown to be correlated to the distribution of the WSS. (Kittaka et al., 2020). However, due to the limited size of the micro vessels and the poor resolution of the diagnostic techniques, a proper analysis of the local hemodynamics and microvascular perfusion impairment has yet to be reported. Furthermore, there are no biomechanical markers of CMD. However, microvascular resistance can be indirectly evaluated on the basis of perfusion changes (Mathew et al., 2020). Low-resolution positron emission tomography (PET) measurements indicate that cardiac perfusion changes locally (El-Tallawi et al., 2020). Indeed, from a hemodynamic standpoint, a sensitive variation in the distribution of velocity gradients (and therefore WSS) should take place.

**FIGURE 1:**
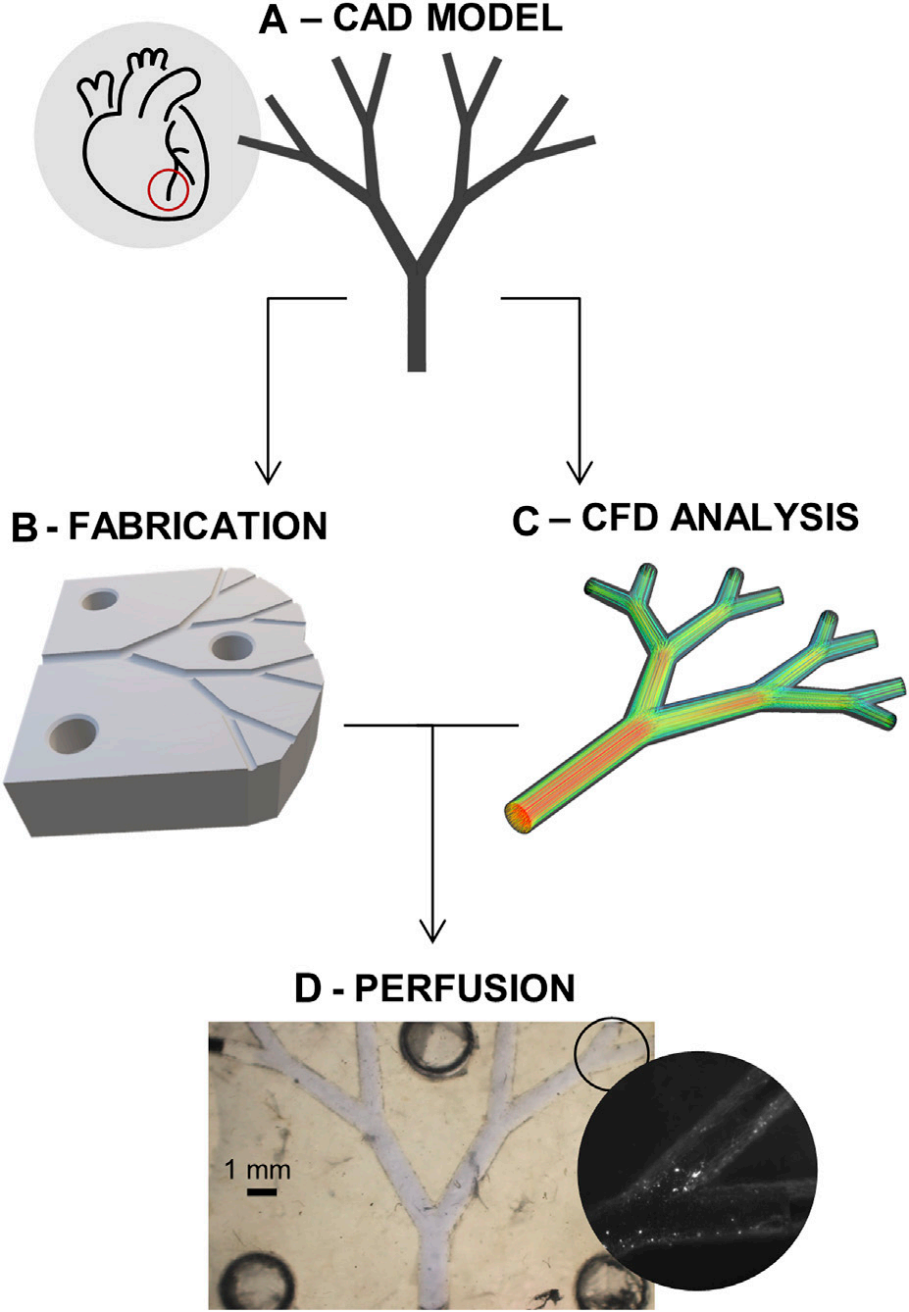
Project schematic. **(A)** CAD model of the idealized microvascular system. **(B)** Fabrication of the microfluidic devices. **(C)** Computational fluid dynamics (CFD) analysis of system. **(D)** Coupling of the *in-vitro* perfusion and the *in-silico* model for improved quantification of the flow impairment.

Since knowledge of hemodynamic changes in impaired microcirculation is scarce, we herein propose an integrative platform able to quantify changes in local hemodynamics occurring during microvascular perfusion impairment. The platform integrates a perfusable, biocompatible fluidic device with *in-silico* analysis. This conceptual workflow, as depicted in Figure 1, provides configurable control of occlusions, and allows quantification of the changes in local hemodynamics and perfusion. The fluidic platform is fabricated by additive manufacturing methods, with the resulting *in-vitro* information being used as an input to create *in-silico* models of local hemodynamic biomarkers of microvascular impairment. Rather than establishing an *in-vivo* biomechanical descriptor of CMD, this work aims to improve CMD diagnostics in two aspects. First, the fluidic platform is biocompatible, and thus allows the investigation of cellular changes in response to local hemodynamic stimulation. Second, since the computational investigation is based on idealized microvascular geometries, it can be easily adapted to patient-specific scenarios.

## 2 Materials and methods

To allow definition of a model for microvascular impairment through the control of downstream obstructions, work was subdivided into two phases, where the same “idealized” model of coronary microcirculation was employed (Figure 1A). First, a fluidic platform made from biocompatible substrate materials was fabricated and used to extract appropriate hemodynamic boundary conditions (Figure 1B). Next, after the creation of a suitable *in-silico* model, CFD simulations of the same platform were conducted for both unobstructed and obstructed networks (Figure 1C,D).

### 2.1 Idealized coronary model

To model coronary microvasculature and analyze hemodynamic changes occurring in the coronary microvasculature, the size of the pre- and arterioles was selected. Here, the choice represents a compromise between building patient-specific models of a portion of the large microvasculature, similar to recent work by Morris and co-workers, Morris et al. (2020) and building more idealized but denser surrogate network models of the small microvasculature, as previously shown in Köppl et al. (2020). Here, our focus is to create a coupled experimental and computational platform to quantify hemodynamic changes occurring in the small microvasculature at the level of the larger epicardial arteries, and thus we chose a microvascular tree model comprising sequential bifurcations. In brief, the model was built considering three primary parameters: 1) the length of the vessels in each bifurcation, 2) the radius of the arterioles and 3) the angle of the bifurcation. To this end, Murray’s law of radius bifurcation (Revellin et al., 2009) was followed,

$${\left(radius\;of\;parent\;vessel\right)}^{3}=\sum {\left(radius\;of\;child\;vessels\right)}^{3}$$

Much of the branching vasculature in mammalian circulatory systems obeys Murray’s law. When obeyed, there exists a functional relationship between vessel radius and volumetric flow, average linear velocity, Reynolds number and the pressure gradients in individual vessels. In the current study, the total in-to-out length of the fluidic structure is 1 cm. The geometrical model was designed using Solidworks (Dassault Systems, Waltham, MA, United States). Importantly, the microvascular tree model with an inlet diameter of 1 mm and an outlet diameter of 0.4 mm was suitable for manufacture using 3D printing, as will now be discussed.

### 2.2 *In-vitro* model of coronary microcirculation

#### 2.2.1 Chip fabrication

The defined model of the coronary microcirculation was used to design fluidic devices for *in-vitro* analysis of perfusion impairment. As previously noted, a primary aim of the current study was to demonstrate a simple manufacturing process for the generation of biocompatible fluidic devices for biochemical analyses. Accordingly, a range of material features were deemed important. (Scott and Ali, 2021; Khosla et al., 2022). These included fabrication cost, structure resolution, device repeatability and biocompatibility.

**FIGURE 2:**
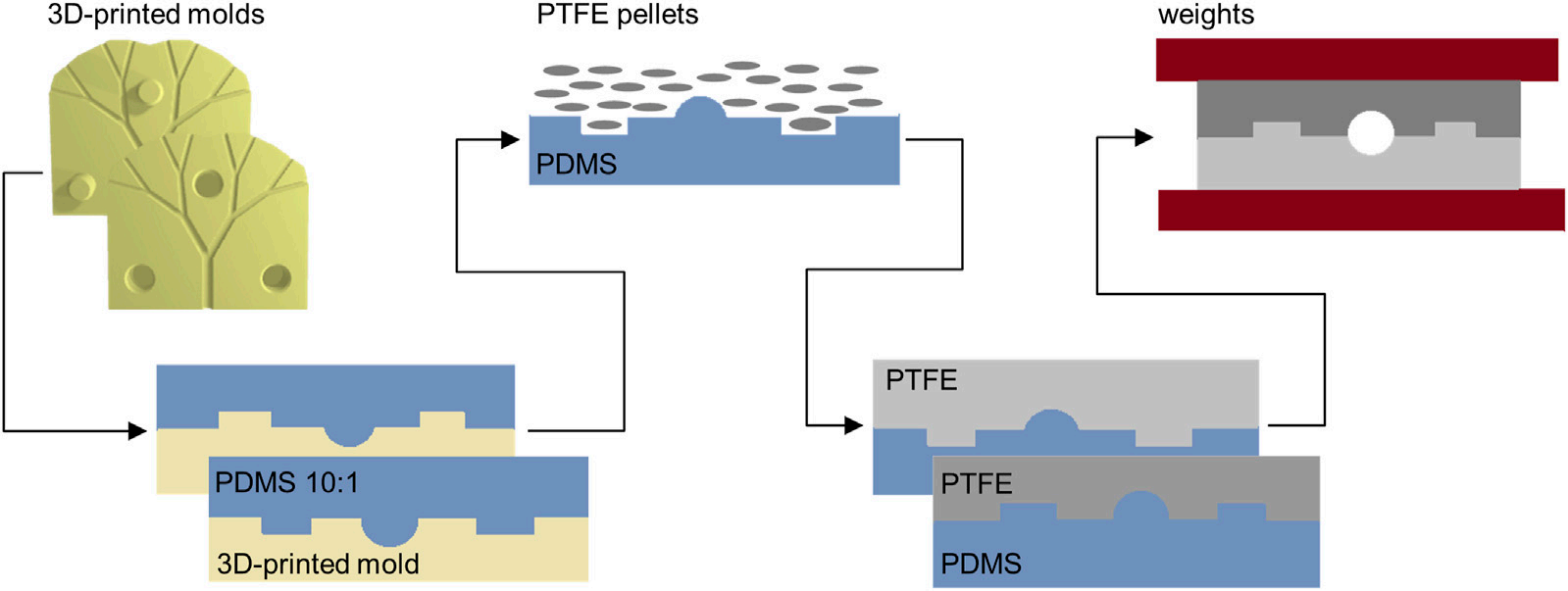
Chip fabrication. The CAD model of the vascular system is used to fabricate, via additive manufacturing, 3D-printed molds. The two-sided molds are then used to obtain PDMS negative molds. The PDMS devices can tolerate the high temperatures needed to melt PTFE pellets. Finally, the two sides of the Teflon™ devices are sealed together simply by heating at an intermediate temperature.

To allow the creation of fluidic channels with circular cross sections, additive manufacturing techniques were employed instead of more conventional photolithographic and/or soft lithographic methods. (Sun et al., 2020; Prabhakar et al., 2021). Specifically, 3D printing and injection molding were considered to be ideally suited for the current study. Additionally, and based on the need to ensure biocompatibility and cell-seedability, polytetrafluoroethylene (PTFE or Teflon™) was chosen as the preferred substrate material. (Ren et al., 2011). As shown in Figure 2, 3D-printed molds were used to fabricate Teflon™ devices. To ensure easy alignment of the two-halves of final device, molds integrated either holes or pillars. Master molds were printed in acrylonitrile butadiene styrene (ABS) using a ProJet MJP 2500 Plus printer, presenting a X-Y-Z resolution of 32 µm. ABS is a thermoplastic polymer, and typically used in injection molding since it has a melting temperature of 150°C. However, since the fabrication of Teflon™ devices requires heating to temperatures above 175°C, an intermediate step incorporating the fabrication of polydimethylsiloxane (PDMS) molds introduced. Details are provided in Supplementary Section 1.1. Briefly, using a ratio of 10:1, PDMS was cured to form negative molds. The negative PDMS molds were then used to cast the Teflon™ halves that form the final device. This was achieved by melting Teflon™ pellets at 200°C for 2 h. After the Teflon™ halves had cooled to room temperature, they were aligned using the hole-pillar system; needle tips were introduced in the formed circular holes. Finally, the entire construct was clamped together and heated up to 145°C for 2 h to ensure sealing and the formation of the enclosed channel network. The resulting fluidic device was then ready to be used for perfusion and/or biocompatibility studies, following sterilization through autoclaving and UV light exposure. Detailed protocols and additional information regarding the fabrication of Teflon™ devices is provided in the Supplementary Section 1.1.2. (Ren et al., 2011; Ren et al., 2014).

#### 2.2.2 Repeatability analysis

The developed protocol for the fabrication of Teflon™ devices was evaluated in terms of feature reproduction and reproducibility before and after sealing of the Teflon™ halves. First, to assess the quality of the fluidic channels and the congruence of the Teflon™ chip after fabrication, surface profiles were measured using a 3D laser scanning microscope (Keyence VK-X100) and processed through the VK Analyzer software. Surface profiles of the 3D-printed molds, the PDMS molds and the final Teflon™ chip halves (before the sealing) were measured. By selecting three regions of interest, repeated measurements of the channel width were performed (Supplementary Section 1.3.1). ImageJ (https://imagej.net/) was used to assess the widths of the channels in the different structures. Specifically, for each image, the width of the central channel, the left and the right bifurcating vessels were measured (Supplementary Figure S5). Non-parametric comparisons through Mann-Whitney U-tests were conducted between corresponding regions of interest of the Teflon™ and PDMS structures. To verify uniform sealing of Teflon™ devices, images of the complete chips were made using a stereo microscope (SMZ1000, Nikon). Additionally, a high-speed camera (either a Hamamtsu C11440 or MotionPro Y5) was used to monitor the flow of 1-µm diameter fluorescent polystyrene beads through the channel network. These movies were used to qualitatively assess any leakages along the entire flow path.

#### 2.2.3 Biocompatibility analysis

Before seeding cells for a preliminary biocompatibility assessment, Teflon™ devices were sterilized. This was achieved by placing microfluidic devices into sterilization pouches and autoclaving at 121°C for 8 min wet and 15 min dry. The autoclaved packets were then placed in a 60°C oven for 2 h, to ensure complete drying. Finally, the packages were exposed to UV light for 1 h, and then opened under a biological cabinet hood. Human Embryonic Kidney 293 cells (293T Flp-in T-REX, HEK cells) were used to seed the devices. The cells were cultivated in T75 flasks until full confluence was reached, by employing a culture medium of Dulbecco’s Modified Eagle Medium (DMEM) solution, with 10% fetal bovine serum (FBS) and 1% penicillin-streptomycin (to prevent contamination). To seed cells within the devices, 5 mL of Trypsin was added. After 3 min of gentle shaking, PBS was added to neutralize the Trypsin, and the cell density of the obtained suspension was measured. A final seeding density of 3 × 10^4^ cells/mL was chosen. Next, the cell suspension was centrifuged; the cell pellet was isolated; and re-suspended in appropriate culture solution to meet the final seeding density.

To facilitate cell seeding, Teflon™ tubes were connected to the needles introduced in the chip inlet and outlets. 1 mL syringes having an outer diameter of 0.63 mm were connected to the tubes to create resistance and ensure that medium and cells could not outflow the channels. First, only the culture medium (DMEM supplemented with 10% FBS and 1% penicillin-streptomycin) was introduced in the microfluidic device. Next, 0.1 mL of HEK cells were delivered using a 1 mL syringe. After optical verification of correct cell introduction, devices were placed in an incubator (set at standard criteria of temperature 37°C, relative humidity 95%, and CO_2_ 5%). The medium was exchanged every 2 days. On day seven, the number of cells in the microfluidic devices was determined via flow cytometry measurements. Specifically, 0.25 mL of Trypsin was added into the channels. After gentle shaking, Trypsin exposed cells were removed with the syringe and collected in an Eppendorf tube. The same amount of PBS was then added to the Eppendorf tube to neutralize the Trypsin. Finally, 0.1 mL of the solution containing resuspended cells was used for cell counting and flow cytometry characterization (CytoFLEX S, Beckman Coulter). Details are provided in the Supplementary Section 1.3.2.

### 2.3 Development of the *in-silico* model

**FIGURE 3:**
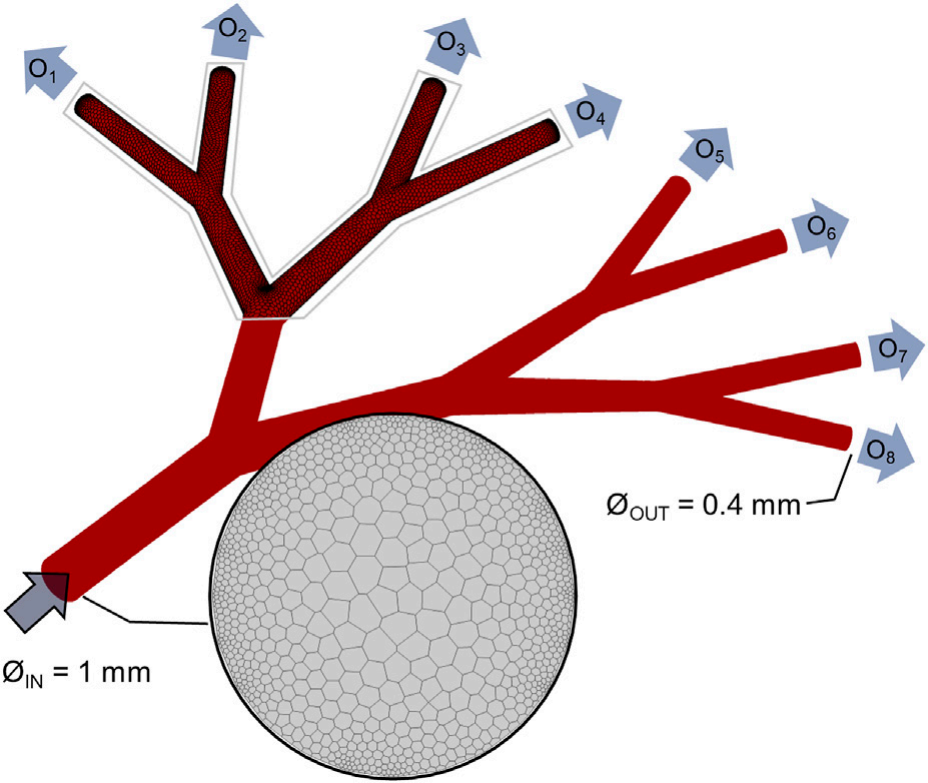
Computational model. Geometrical model of the idealized vascular system, with one 1-mm diameter inlet and 8 identical outlets (named O_1_, …, O_8_) with 0.4-mm diameter. Details of the polyhedral computational mesh are given for the inlet and portion of the wall.

The CAD model of the coronary microcirculation was used to create the CFD model, as shown in Figure 3. To perform *in-silico* analysis, the geometry of the microvascular system was discretized into polyhedral elements. (Sosnowski et al., 2018). Following standard numerical practice, the choice of the final mesh size (223′995 cells) was made after a grid-independence study (Supplementary Section 1.2). A number of assumptions were made when defining the flow regime and the model boundary conditions. First, fluids were considered to be Newtonian, and water properties (density and viscosity) were imposed to the model, since water was employed in the *in-vitro* analysis instead of blood. Based on a maximum Reynolds number of 0.018, all flows were considered to be laminar. Second, steady-state CFD simulations were preferred, since the pulsatility of the blood flow in the arteriolar component of the microcirculation was neglected. Indeed, it is known that pulsatile flow is transmitted to the microvascular bed, and after massive damping becomes a relatively continuous venous flow (Pan et al., 2022). Third, and as previously noted, *in-vivo* quantification of the local hemodynamics is difficult, due to the poor resolution of existing techniques. For better coupling of the *in-vitro* and *in-silico* models, constant boundary conditions were imposed to the *in-silico* model. Precisely, a constant flat velocity of 0.012 m/s was imposed at the inlet section. (Ghoshhajra et al., 2021). The outlet boundary conditions, consisting of flow out-split, were defined based on experimental measurements, as will be discussed in Section 3.2. The computational model and the analyses were conducted in ANSYS Fluent (v. 2022R1, Ansys Inc., Canonsburg, PA, United States), with the solver settings being detailed in Table 1. Hemodynamics was analyzed in terms of velocity, pressure field, and the WSS distribution.

**TABLE 1:** List of the solver settings.

Type	ANSYS fluent—pressure-based
Pressure-velocity coupling method	Coupled
Flux type	Rhie-Chow: momentum-based
Spatial discretization scheme—gradient	Least squares cell based
Spatial discretization scheme—pressure	Second order
Spatial discretization scheme—momentum	Second order upwind
Scheme	Pseudo time method
Warped-face gradient correction	
Pseudo time explicit relaxation factors	
Pressure	0.5
Momentum	0.5
Density	1
Body forces	1
Residuals	10^–5^

## 3 Results

### 3.1 The fluidic device

**FIGURE 4:**
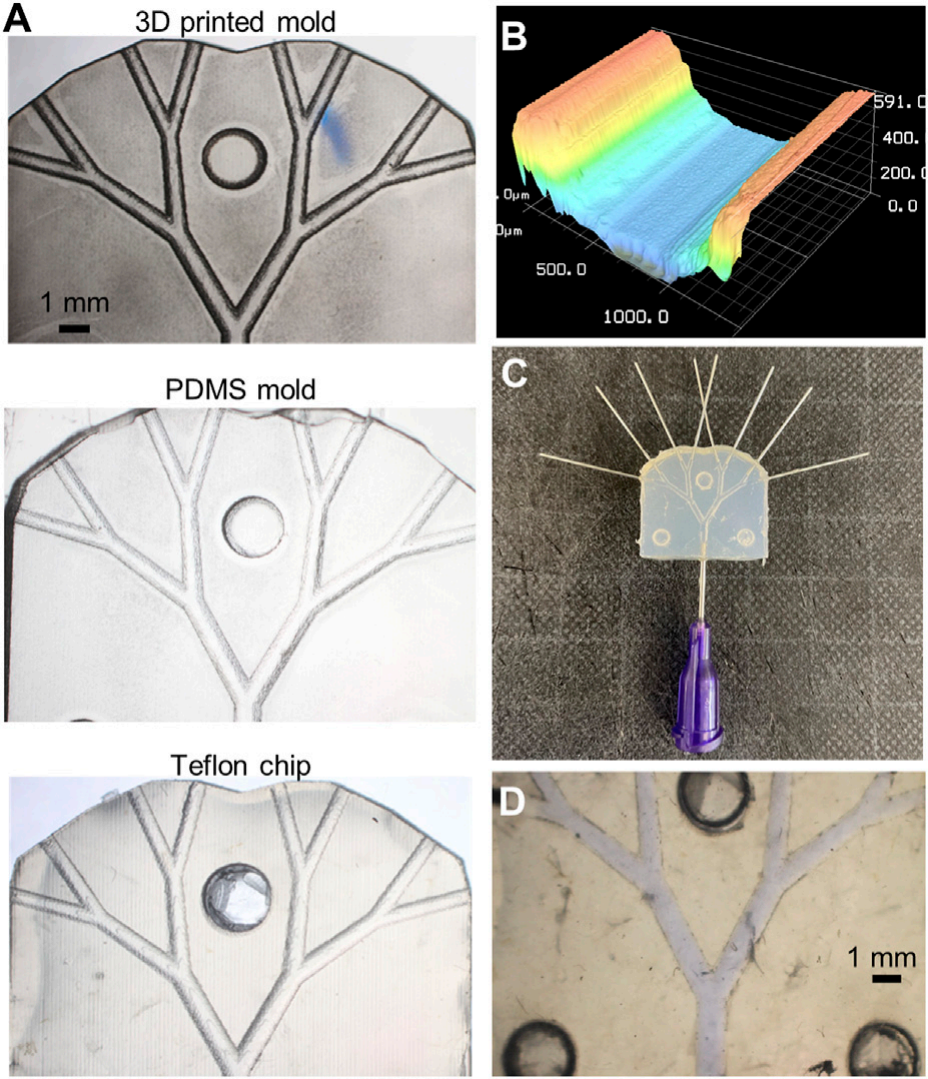
Fabricated platform. **(A)** Stereoscope images of the original 3D-printed mold used to obtain the Teflon™ device (on the right). **(B)** Profilometry measurement of microfluidic channels, where the typical structure or 3D-printed layers are observable. **(C)** Sealed Teflon™ device with needle tips fixed at the inlet and outlets. Stereoscope image of the sealed chip containing a blue-ink solution to visualize fluidic leakage. **(D)** Stereoscope image of the sealed chip containing a blue-ink solution to visualize fluidic leakage.

As previously noted, a critical aspect of the fabrication processes was the ability to create microscale features in a reproducible fashion. Since fabrication involved three component steps, the separated halves of the 3D-printed molds, the PDMS structures, and the resulting Teflon™ devices (Figure 4A) were prepared for the surface profile measurements. Specifically, fabrication consistency was tested by making 5 sets of channels, for each of which 4 microfluidic devices were created (Supplementary Section 1.3.1). The PDMS halves were separated easily from the 3D-printed negative molds and reproduced the microscale features of the master molds. Similarly, the Teflon™ pellets melted into identical shapes. Although minor variations in the hollow and pillar structures were observed, these did not impact the correct alignment of each half. A consistent geometrical pattern was observed in all the analyzed structure, as visible from the surface profile shown in Figure 4B. The channel curvature did not change, with the radius of the curvature being identical to that defined in the 3D-printed mold. Surface roughness, which was observed in all devices, was a result of imperfections in the 3D-printed mold. After sealing, Teflon™ devices were ready for perfusion experiments (Figure 4C). Optical evaluation of the containment of ink solutions (Figure 4D) was initially performed by stereo-microscopy to assess fluid integrity of microfluidic devices. The devices functioned as expected and did not leak. Accordingly, the sealed fluidic devices were ready to be employed for the biocompatibility and perfusion impairment analyses.

**FIGURE 5:**
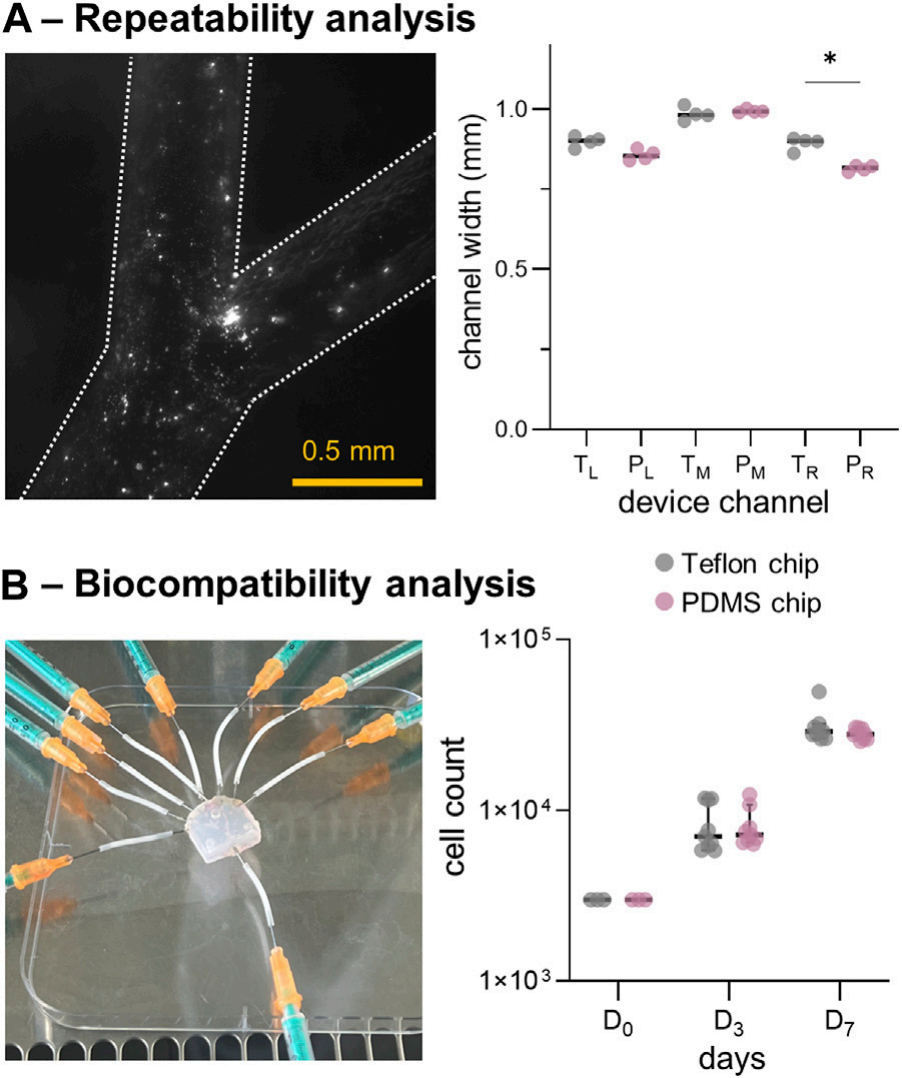
*In-vitro* model repeatability and biocompatibility results. **(A)** Left: fluorescence microscopy images of a fabricated Teflon™ device. Right: comparison of channel widths for the Teflon™ (T) and PDMS (P) devices. **(B)** Left: Teflon™ device platform employed for seeding HEK cells. Right: results of the cell count at days 1, 3 and 7, cultured in a Petri-dish presenting the fabricated Teflon™ and PDMS materials. *: *p*-value <0.05.

Following the verification of the channel patency through stereo-microscopic imaging, sealing was assessed through qualitative fluorescent imaging (Figure 5A, left panel). As can be seen, the 1-µm polystyrene beads were perfectly contained within the chip during flow. Four Teflon™ devices (and their PDMS molds) were used to assess fabrication repeatability. As shown in Figure 5A and Supplementary Figures S5–S8, the channel width at different positions in the flow path (for both Teflon™ and PDMS devices) varied by a maximum of 20 µm. Due to minor fabrication inconsistencies, some structural differences were observed in certain locations, most notably the right Teflon™ and PDMS channels (*p*-value: 0.029). Despite the symmetric configuration of the 3D-printed molds, slight but significant differences (*p*-value: 0.029) were found between the left and right PDMS channel widths (Supplementary Table S3). No significant differences were found between the left and right channels in the Teflon™ devices. To summarize, profilometry measurements confirm the robust generation of Teflon™ microfluidic devices, integrating circular cross-sectional channels.

We then assessed the biocompatibility of Teflon™ devices by growing HEK cells within the fluidic network. As shown in Figure 5B (left panel), the sterilized Teflon™ platform was connected to the syringes containing DMEM for cell seeding. First, material cytotoxicity was assessed by seeding HEK cells in standard Petri-dishes containing Teflon™ or PDMS scaffolds (flat round layers with area of ∼3.5 cm^2^ fitting the 12-well cell culture plate). Importantly, HEK cells continued to proliferate when exposed to both Teflon™ and PDMS over a period of 7 days (Figure 5B). Comparison of cell harvests at day 3 (D_3_) and 7 (D_7_) indicated no statistical difference between the two fabricated materials, confirming similar levels of toxicity. However, flow cytometry measurements showed the presence of more apoptotic HEK cells as compared to the literature and control HEK culture (Supplementary Figure S8). This is because in real studies we would need to add continuous perfusion (instead of the static cell culture conditions assumed here) to prevent evaporation of the medium and consequent cell death.

### 3.2 Quantification of perfusion impairment

**FIGURE 6:**
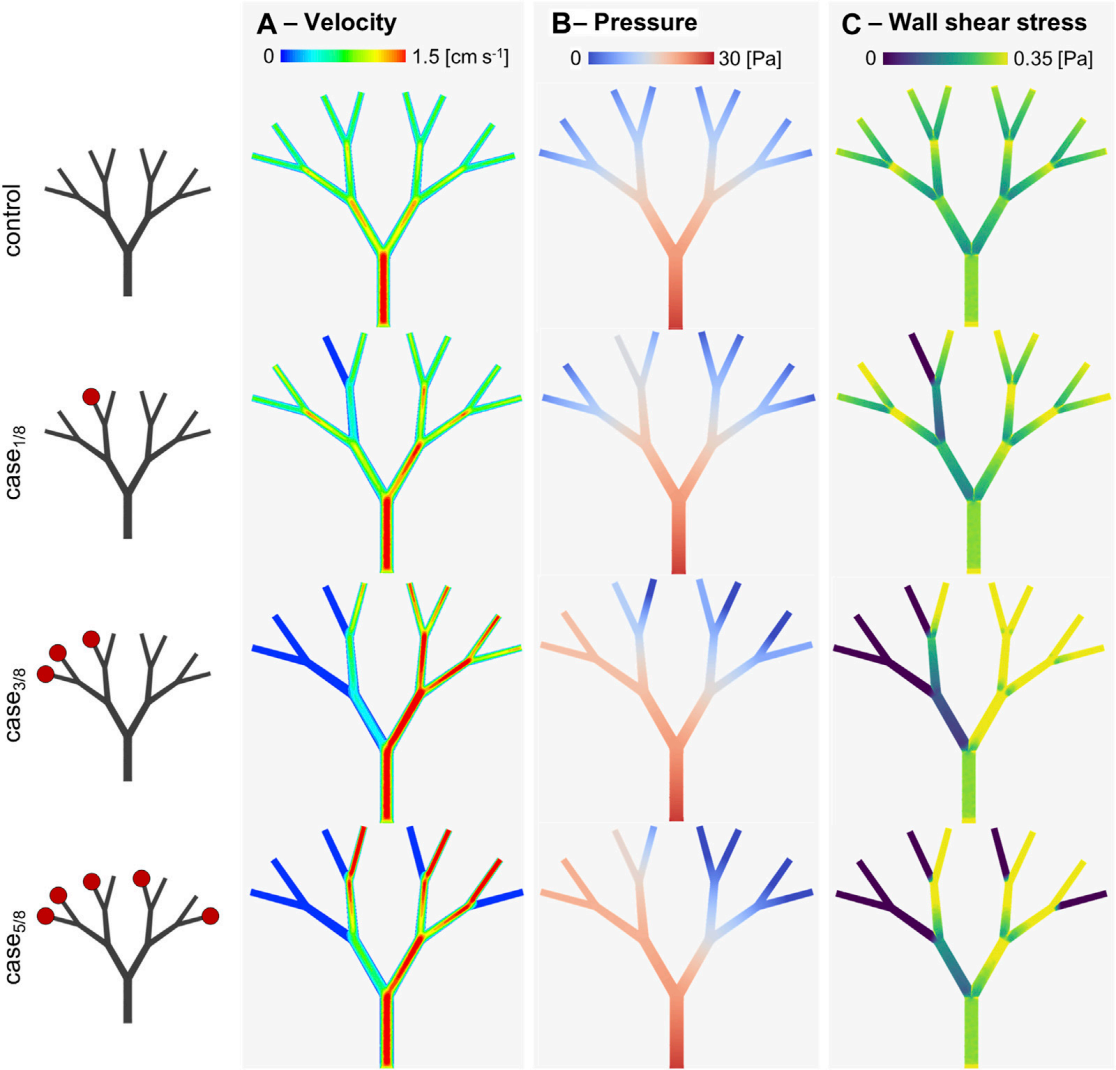
*In-silico* results. **(A)** velocity magnitude, **(B)** pressure and **(C)** wall shear stress distributions for the different considered scenarios. Specifically, the scenarios presenting a different degree of arteriolar occlusion (‘case_1/8_’, ‘case_3/8_’, and ‘case_5/8_’) are derived from the *in-vitro* model, while the control case was used as comparison. The increasing impairment level results in a sensitive change in the WSS distribution, suggesting a compensatory *in-vivo* effect.

The developed fluidic platform was then used to couple *in-vitro* and *in-silico* models. First, the inlet of Teflon™ device was connected to a syringe pump and used to quantify the mass flowrate at various levels of impairment. Briefly, three microvascular obstruction scenarios were represented, with an increase in the degree of impairment constructed in an asymmetric configuration. In addition to a physiological scenario with a homogeneous distribution at the outflow (‘control’), one, three and five of the eight outlets were sequentially blocked, i.e., ‘case_1/8_’, ‘case_3/8_’, and ‘case_5/8_’ respectively. Specifically, device outlets were either connected to Teflon™ tubes that were left open or connected to empty 1 mL syringes to mimic infinite outlet resistance. As depicted on the left side of Figure 6, the location of the occluded vessels defined an asymmetric flow distribution. Hydrodynamic pumping allowed definition of the flow rate imposed at the inlet. To compare perfusion scenarios, the inlet flow rate was set to 195 µL/min. For an experimental run time of 60 s, the fluid volume collected at each outlet was assessed and the out-split quantified. For the ‘control’ scenario, a relative volume fraction of ∼0.125 was found for each branch. This was qualitatively observed with minor variations, as presented in Table 2. The out-split ratios for the obstructed devices are presented in Table 2. Imposing the out-split as boundary conditions, CFD analyses of the four scenarios were performed. Figure 6 presents the computed local hemodynamics, in terms of velocity magnitude (Figure 6A), pressure (Figure 6B) and WSS (Figure 6C) contour maps. Due to the assumption of a flat inlet velocity profile, no differences between control and impaired scenarios were found in terms of velocity magnitude, pressure and WSS distributions in the main channel. As expected, asymmetric distributions of velocity magnitude and pressure were observed along the bifurcating geometry.

**TABLE 2:** Volumetric flowrate quantification of perfusion impairment for the simulated scenarios with the *in-vitro* platform. The outlet flowrates, normalized with respect to the total computed flowrate, are set as flow out-splits for the *in-silico* model.

	control		case_1/8_		case_3/8_		case_5/8_	
	Flowrate (·10^–6^ m^3^/s)	Out-split (a.u.)	Flowrate (·10^–6^ m^3^/s)	Out-split (a.u.)	Flowrate (·10^–6^ m^3^/s)	Out-split (a.u.)	Flowrate (·10^–6^ m^3^/s)	Out-split (a.u.)
Inlet	3.01	1.00	2.95	1.00	3.65	1.00	4.74	1.00
O_1_	0.375	0.125	0.36	0.12	0	0	0	0
O_2_	0.380	0.126	0.46	0.16	0	0	0	0
O_3_	0.387	0.129	0	0	0	0	0	0
O_4_	0.378	0.126	0.41	0.14	0.66	0.18	1.5	0.32
O_5_	0.365	0.121	0.45	0.15	0.75	0.21	0	0
O_6_	0.369	0.123	0.41	0.14	0.75	0.21	1.58	0.33
O_7_	0.374	0.124	0.41	0.14	0.83	0.23	1.66	0.35
O_8_	0.379	0.126	0.45	0.15	0.66	0.18	0	0

Table 3 reports the area-weighted averaged WSS results quantified in each model branch. It should be noted that WSS is highly sensitive to changes in the local hemodynamics. Starting at the first bifurcated vessels (the C_2_ level), significant changes in WSS were observed, not only as a function of the occluded vessels following the same bifurcating line, but also in parallel branches. This is evident when comparing the WSS value of the C_2_-B_1_ branches in ‘case_3/8_’ and ‘case_5/8_’. Despite having the same distribution of occluded vessels on the left-hand side (i.e., O_1_, O_2_ and O_3_), ‘case_3/8_’ exhibits a WSS value approximately half observed in ‘case_5/8_’. This suggest that there may be a compensatory phenomenon, as expected in real microcirculations, where the few unobstructed vessels are expected to receive higher flowrates to supply the cardiac tissue with the necessary nutrients and oxygen.

**TABLE 3:** Results of the *in-silico* analyses in terms of area-weighted averaged wall shear stress computed for each branch of the model. For each branch, the channel (C) and the bifurcated vessel (B) are sequentially labeled, as shown in the graphic on the right hand side. The highest values are colored red, while the lowest are colored blue.

Area-weighted averaged wall shear stress (Pa)					
	control	case_1/8_	case_3/8_	case_5/8_	
C_1_	0.36	0.36	0.36	0.36	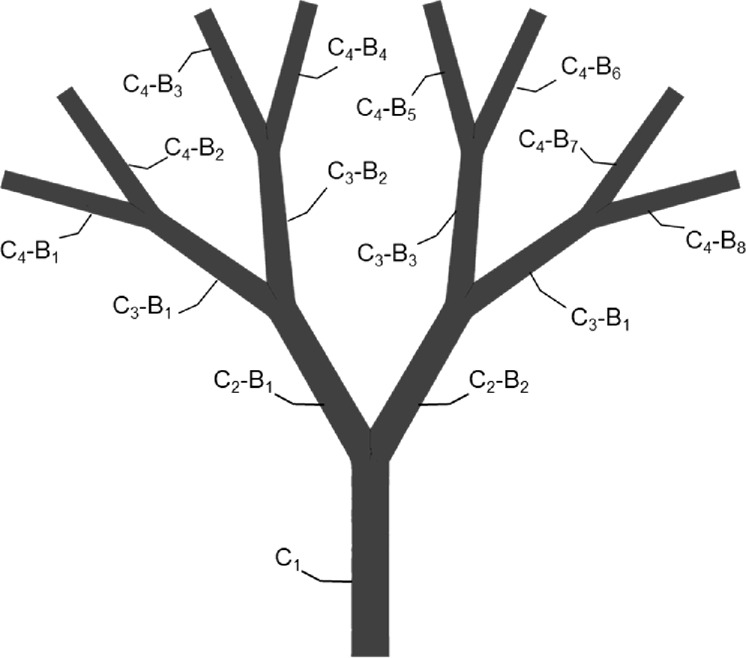
C_2_-B_1_	0.24	0.21	0.09	0.16	
C_2_-B_2_	0.24	0.28	0.40	0.33	
C_3_-B_1_	0.27	0.30	0.00	0.00	
C_3_-B_2_	0.27	0.15	0.19	0.34	
C_3_-B_3_	0.27	0.31	0.45	0.35	
C_3_-B_4_	0.26	0.30	0.43	0.37	
C_4_-B_1_	0.28	0.27	0.00	0.00	
C_4_-B_2_	0.28	0.36	0.00	0.00	
C_4_-B_3_	0.26	0.00	0.01	0.01	
C_4_-B_4_	0.26	0.29	0.37	0.67	
C_4_-B_5_	0.26	0.32	0.44	0.02	
C_4_-B_6_	0.26	0.30	0.44	0.69	
C_4_-B_7_	0.28	0.31	0.51	0.78	
C_4_-B_8_	0.28	0.33	0.40	0.00	

## 4 Discussion

Several studies have demonstrated the association of CMD with a worsening prognosis. This condition leads to a reduction in the quality of life due to angina-like symptoms that are caused by impaired myocardium perfusion. (Sucato et al., 2020; Spione et al., 2022). The challenge in understanding perfusion impairment in the microvasculature represents a common roadblock in predicting disease progression. Herein, we have presented a conceptual workflow to evaluate the hemodynamic changes occurring in the coronary microvasculature during perfusion impairment. Our platform integrates the *in-vitro* modification of the local hemodynamics and the *in-silico* quantification of velocity and pressure gradients. This workflow is achieved by using the fluid conditions measured on perfusable, biocompatible fluidic chip as boundary conditions for subsequent CFD analyses.

Starting from *in-vitro* hemodynamic modelling, our first aim was to develop a methodology to fabricate microfluidic devices with high reproducibility and characterized by easy manufacturability/freedom of microchannel structure. For instance, obtaining circular cross-section of the microchannels represented an essential feature of our experimental methodology, given that the endothelium of the blood vessels is proved to be delicately reacting to the distribution of shear stress. (Roux et al., 2020). The second aim was to employ a substrate material that, in addition to be easily structured, offers optimal conditions of cyto-compatibility. To meet these objectives, we fabricated the microfluidic devices using both additive manufacturing and Teflon™-based soft lithography. In regard to the microchannel structure, our approach allows the generation of configurable fluid networks (based on a geometrical tree model of the coronary microcirculation) in a rapid and robust manner. In the past decade, PDMS has emerged as the dominant biomaterial used in the fabrication of microfluidic platforms. (Xia and Whitesides, 1998; Fiddes et al., 2010; Yang et al., 2018). By leveraging other methods, such as mechanical microscale milling of metals to create master molds for PDMS fabrication, Wilson et al. (2011) or re-shaping through silica sol-gel reactions, Jia et al. (2008) circular cross-section microchannels instead of the standard rectangular ones can be obtained. However, these techniques are unable to effectively reproduce gradual changes in the diameter of the channel, unlike 3D printing. By combining additive manufacturing and the repeatable fabrication techniques reported in this work, we were able to generate complex structures comprising circular-cross section microchannels. In regard to cyto-compatibility, we verified that the fabricated devices were suitable for cell culture studies under variable perfusion conditions. Even though the cytotoxicity levels between Teflon™ and PDMS devices in static culture conditions were comparable, PDMS devices are known to be characterized by higher gas permeability and molecular absorption when compared to Teflon™ microfluidic networks, which present hydrophobic surfaces. (Yang et al., 2018; Nightingale A et al., 2020; Hess et al., 2021). Since dynamic flow conditions facilitate cell detachment from the channel walls, surface treatments and use of products such as fibronectin can notably improve cell adherence. Indeed, even though we did not directly test dynamic culture conditions, more extensive studies have previously assessed the cyto-compatibility of Teflon™. (Ren et al., 2011; Wang et al., 2018; Akther et al., 2020). Finally, it is noteworthy that the additive manufacturing step poses a limitation in the size of the fabricated microchannels, depending on the resolution of the 3D-printer used. Accessing smaller length scales is possible, at the expense of a long fabrication times to produce large area networks. Furthermore, the mechanical properties of the microvessels were not taken into account, and vessel wall compliance was neglected.

The *in-silico* model developed is potentially able to provide quantitative estimation of hemodynamic changes for different levels of the microvascular geometry. Since the quantification of *in-vivo* hemodynamics in the coronary microvasculature cannot be measured, the use of an *in-silico* model allows imposition of boundary conditions measured through experiment. Steady-state CFD analysis was successful in providing hemodynamic descriptors such as WSS at the level of small microvascular vessels. As previously shown, the local hemodynamics plays a key role in nonobstructive coronary artery disease. (Kumar et al., 2018; Kittaka et al., 2020). That said, current literature assessing the relationship between near-wall hemodynamic alterations (i.e., abnormal WSS patterns) and microvascular alterations is poor. Accordingly, our platform provides a first step in expanding knowledge on the relationship between biomechanical quantities (such as WSS) and clinical measurements (such as coronary flow reserve). To conclude, our integrated platform is suitable for analyzing hemodynamic changes occurring in the coronary microvasculature. The combination of additive manufacturing, microfluidic experimentation and CFD analysis, provides an accessible and cost-effective solution to enhancing the biomechanical understanding of CMD through rapid and noninvasive investigations.

## Data Availability

The raw data supporting the conclusion of this article will be made available by the authors, without undue reservation.
