# Supplementary material for: Integrative modeling of hemodynamic changes and perfusion impairment in coronary microvascular disease
Source: Front Bioeng Biotechnol. 2023 Jul 26;11:1204178. doi: 10.3389/fbioe.2023.1204178 (PMC10410158; doi:10.3389/fbioe.2023.1204178)
Supplement: Supplementary file 1 [file DataSheet1.docx]

Supplementary Material

Integrative modeling of hemodynamic changes and perfusion impairment in coronary microvascular disease

Monika Colombo*, Palak Chaudhry^†^, Yvonne Oberholzer^†^, and Andrew J deMello

*** Correspondence:** [mc@mpe.au.dk](mailto:mc@mpe.au.dk)

† These authors contributed equally to this work

**Table of content:**

1. Supplementary Data
   1. Fabrication of the fluidic chip
      1. Comparison to PDMS
      2. Teflon^TM^ properties and detailed protocol
   2. Grid independence analysis
   3. Repeatability analysis
      1. Surface profile results
      2. Biocompatibility
2. Supplementary Figures & Tables
   1. Supplementary Figures
   2. Supplementary Tables
3. References

# Supplementary Data

## Fabrication of microfluidic devices

### Comparison to PDMS

PDMS is the standard material for creating microfluidic devices via replication. The most common replication technique for molding PDMS channels involves using positive or negative resist masters in an SU-8 photoresist. The major limitation of this approach is the fact that rectangular cross-section channels are formed, which are unlike the physiological circular geometries. Moreover, the complexity and high-cost of the production of the photoresist mask make this process unsuitable for rapidly protyping patient-specific configurations.

The use of additive manufacturing allows the reduction of production times, as well as versatility in the design of more complex and tortuous geometries. Advantageous features of PDMS include optical transparency and material elasticity, generally superior to Teflon^TM^ transparency and stiffness. As shown in **Figure S1**, negative 3D-printed molds are used to prepare the PDMS devices. Unlike the protocol for the fabrication of the Teflon^TM^ chips, uncured PDMS can be immediately poured onto the 3D-printed molds. After curing at 70℃ for approximately 2.5 hours, the PDMS halves can be extracted and then subjected to plasma treatment.

### Teflon^TM^ properties and device fabrication

The fabrication of fluidic devices involves replicating a desired circuit or network within a solid material. This can be achieved by either direct manufacturing or replication. Teflon^TM^ shows outstanding inertness to various chemicals and extreme resistance against almost all solvents. Compared to other microfluidic materials; the whole-Teflon^TM^ device has additional advantages, such minimal absorption of small molecules into the substrate material, minimal adsorption of biomolecules onto channel walls, and negligible leaching of residual molecules from the substrate material into the contained fluid. Various adherent cell types have been cultured in the Teflon^TM^ chip and are observed to proliferate well within microfluidic channels (with similar proliferation rates to cells in PDMS channels with the same dimensions). The moderate gas permeability of the Teflon^TM^ also makes it suitable for culturing cells inside microchannels for extended periods of time.(1,2)

The Teflon^TM^ devices are used to validate the *in-silico* model of coronary microvasculature. These biocompatible devices are suitable for cellular adhesion of endothelial cells.(3) As shown in **Figure 2** and **Figure S2** and reported in the following protocol, we fabricated Teflon^TM^ devices using 3D-printed positive molds and PDMS negative molds. Finally, thermostatic pressure was used to seal devices.

*PDMS Positive mold preparation*

1. Cover a circular glass petridish with aluminum foil for casting the PDMS.

2. Use double-sided tape to contact the pillared and holed 3D molds onto the aluminum foil.

3. Put this arrangement in a square petri dish.

4. Pour the PDMS base/curing agent at a ratio 10:1 into a plastic.

5. Close the plastic container and keep it in the degassing chamber for 1 minute with a rotation speed of 3000 rpm to remove gas bubbles present in the uncured PDMS.

6. Pour the degassed, uncured PDMS over the 3D molds. Ensure that fluid is poured close to the surface to achieve a constant laminar flow.

7. Place the square petri dish in a vacuum desiccator for 30 minutes to remove all air bubbles.

8. Place the square petri dish in the oven for 2 hours at 70℃. Remove the petri. dish, let it cool for 30 minutes and peel off the PDMS structure.

*Teflon^TM^ chip preparation*

1. Cut the sides of the PDMS molds.
2. Pour the THV 500 Teflon^TM^ pellets over the mold and place all components into an oven at 200℃ for at least 2 hours (**Figure S3**).
3. Peel the Teflon^TM^ chips off, taking care to not break the PDMS mold.

*Sealing of Teflon chip*

1. Align the two Teflon^TM^ device halves to make complete channels.
2. Place the needle tips in the inlet and outlet holes (**Figure S4**).
3. Tighten the screw clamp with the device within and place it in the oven at 145℃ for 2 hours.
4. Seal the joints of the holes with epoxy and leave the device overnight at room temperature.

## Grid-independence analysis

As described in the main text, the geometrical model of coronary arteriolar circulation was discretized into polyhedral cells, which combine the advantages of hexahedrons and tetrahedrons, i.e. low numerical diffusion and rapid semi-automatic generation. Due to the elevated number of neighboring cells, gradients are well-approximated. Furthermore, the mesh quality is high due to limited sensitivity to stretching when compared to tetrahedrons, improving the accuracy and numerical stability of the model. The use of polyhedral meshes creates a more accurate solution with a lower cell count, and therefore a lower computational time.(4)

Sensitivity analysis of different meshes can be used to select a meshing solution with the best trade-off between accuracy and computational cost. Four different mesh densities were generated for sensitivity analysis. **Table S1** explains the distribution of grid points with a varying range of cell sizes. The wider the range of mesh size, the denser and more precise the grid faces and cells.

Mesh sensitivity was analyzed using four different parameters, namely the maximum velocity, area-weighted average (AWA) WSS, AWA pressure, and the number of iterations (computational efficiency) for the completion of the numeric calculations. The same boundary conditions were applied to each mesh, namely:

Velocity inlet = 0.012 m/s

Equal out-split = 0.125 (for all 8 outlets)

In this work, a residual error of 1e5 was chosen as the limit for accuracy. **Table S2** shows the comparison of all the different mesh parameters. Mesh 2 fulfilled the criterion of a marginal error of less than 1% in all parameters, presenting also a tolerable computational time. Accordingly, the mesh settings of Mesh 2 were considered.

## Repeatability analysis

### Surface profile results

Fabrication of microfluidic channels comprised three stages, namely the 3D printing of molds, the creation of PDMS molds, and the formation of Teflon^TM^ structures (**Supplementary Section 1.1**). The reproducibility criteria used for analyzing the fabrication protocol consisted of feature repeatability and surface quality. As a first step, stereo microscopic images of the replica of the molds of each produced set were compared with each other to investigate the morphological repeatability of the molds (**Figure S5**).

To analyze consistency, images of fabricated molds at every stage of the process were taken and are shown in **Figure S6-A**,**B**. Surface roughness was captured in detail using the 3D optical profilometer at three regions - inlet, outlet and at the second bifurcation.

The following measurement settings were used on the 3D optical profilometer:

- Mode: Transparent (top)

- Area: 1 line

- Quality: High-accuracy

To get a three-dimensional image of the channel, the following measurement settings were used:

- Mode: Surface profile

- Area: Standard

- Quality: High-accuracy

The layered pattern at the surface of the 3D molds was observed on all the PDMS and Teflon^TM^ devices, as seen in **Figure S7**. Last, the results of the width measurements (mean values and standard deviations) of the reference channels of the Teflon^TM^ and PDMS chips were statistically compared through Mann-Whitney U-test analysis, as shown in **Table S3**.

### Biocompatibility results

As shown in **Section 3.1**, the cell count results of the Teflon^TM^ and PDMS biocompatibility were similar. However, to allow a more detailed comparison of the HEK population after cell culture, flow cytometry measurements were compared between the control HEK group (cultured under normal conditions) and the groups exposed to the fabricated materials (namely PDMS and Teflon^TM^). **Figure S8** shows the density plots of the forward vs side scatter height for the three groups. It can be observed that when compared to the control group, more debris and apoptotic HEK cells are found when cells are exposed to the fabricated materials. After observing the devices under the microscope, it was possible to see that the static cell culture induced evaporation of the medium, causing a larger fraction of cells to die.(5)

# Supplementary Figures and Tables

## Supplementary Figures


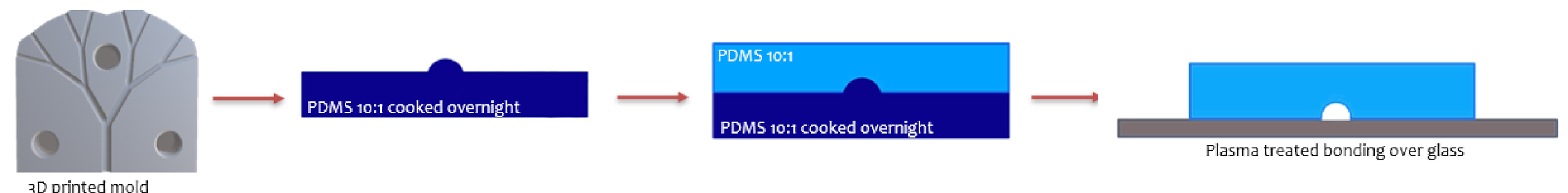


**Supplementary Figure S1:** PDMS fabrication. Process for the fabrication of semi-circular PDMS channels.

**
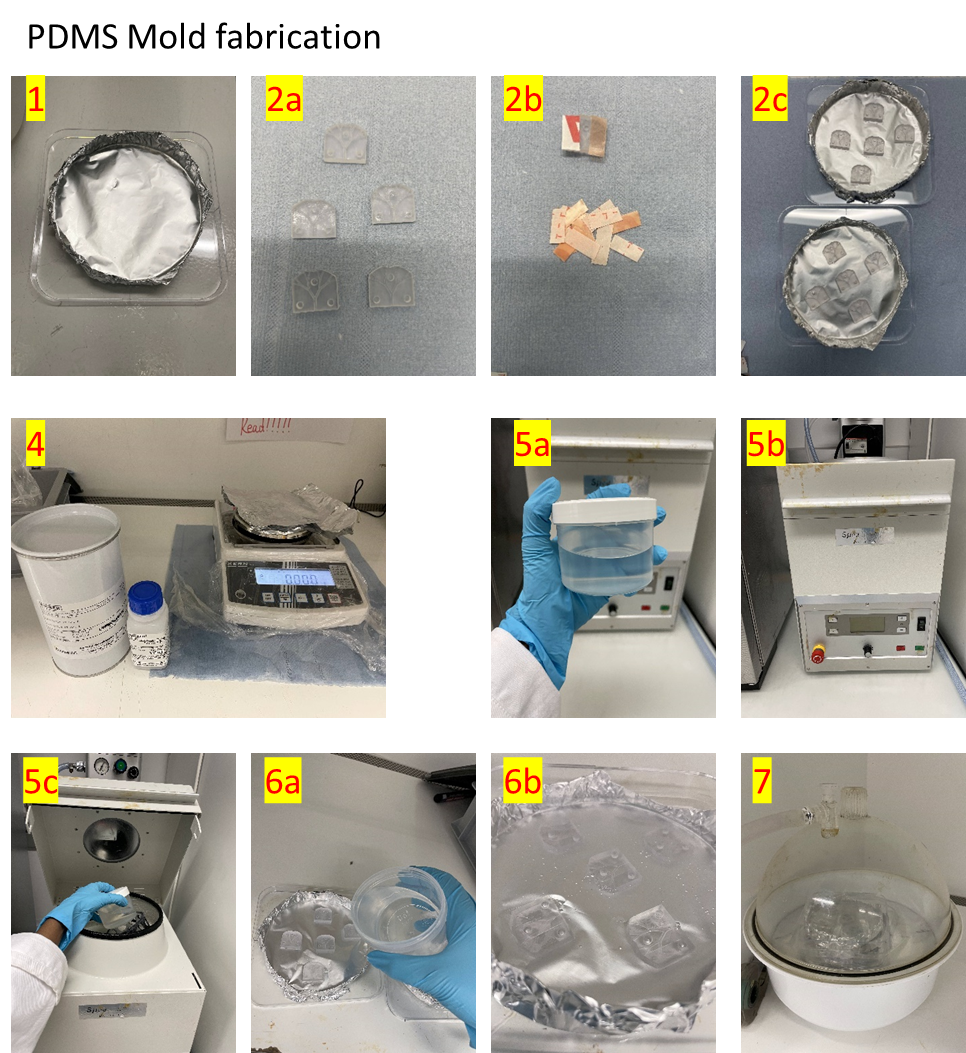
**

**Supplementary Figure S2:** PDMS positive mold fabrication. Process for the fabrication of semi-circular PDMS channels.


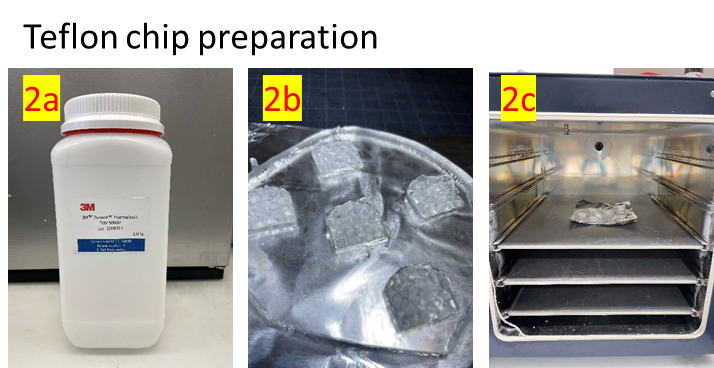


**Supplementary Figure S3:** Teflon^TM^ chip fabrication.


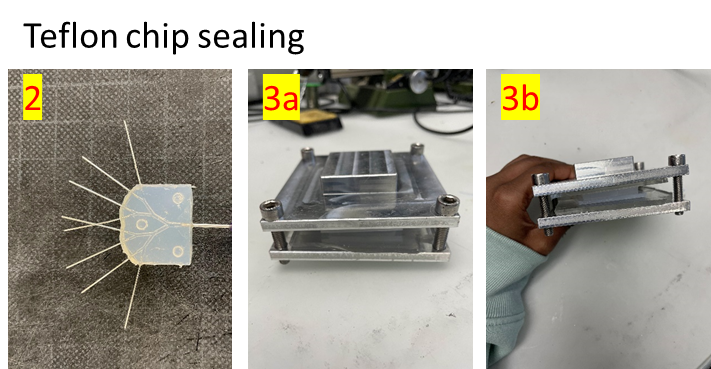


**Supplementary Figure S4:** Teflon^TM^ chip sealing.


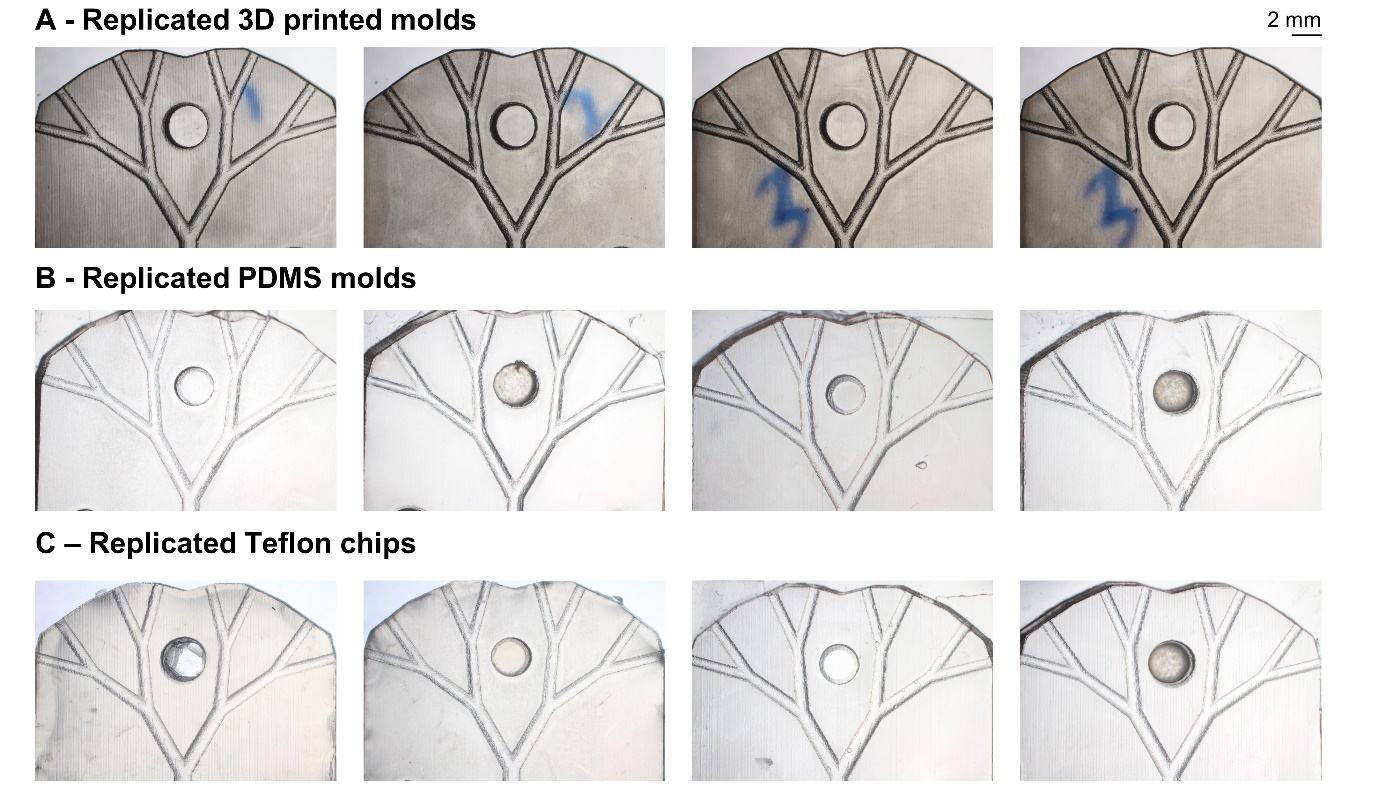


**Supplementary Figure S5:** Consistency of mold at each stage of fabrication and repeatability analysis.


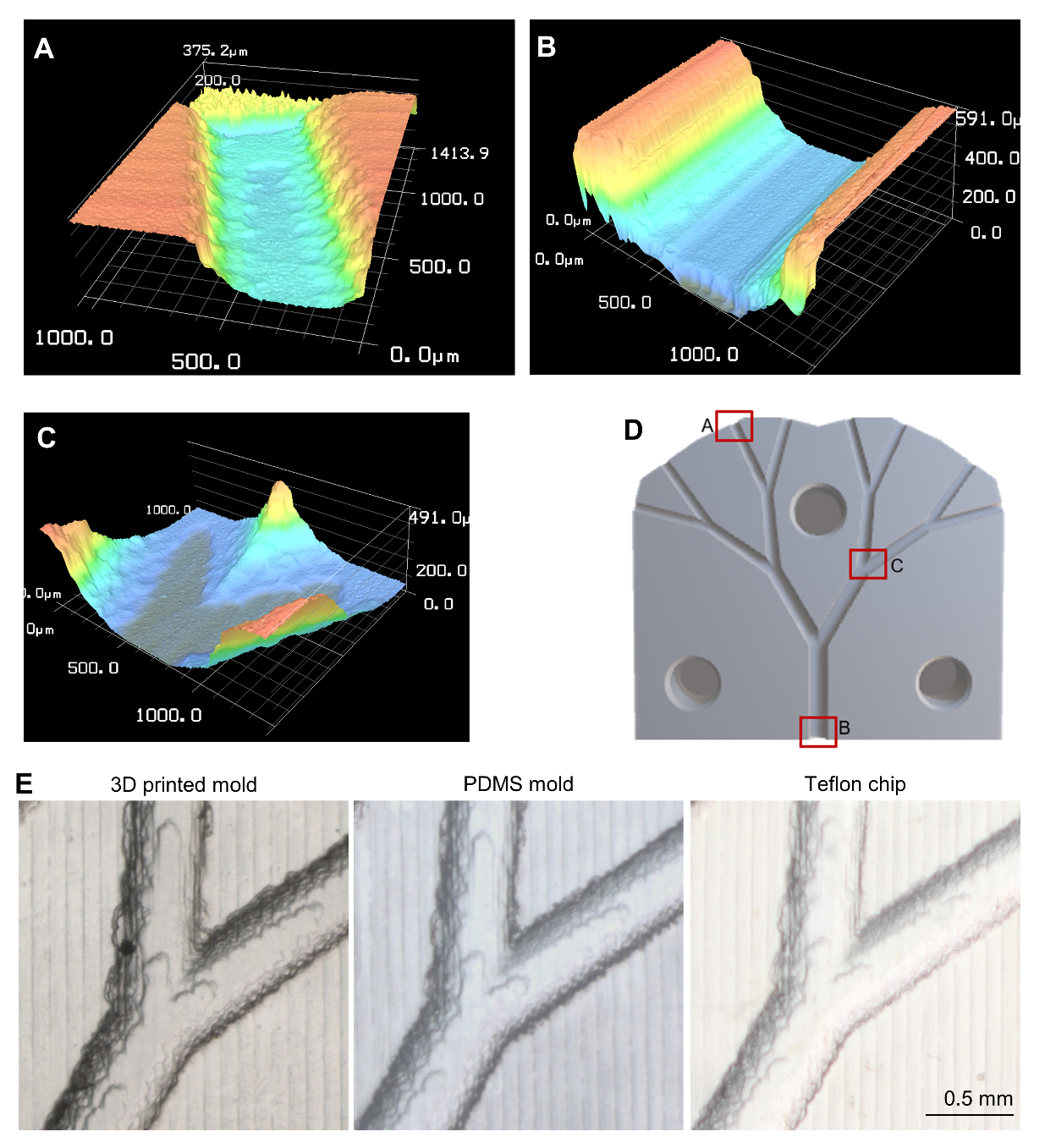


**Supplementary Figure S6:** Different surface profiles of microfluidic channels. **A)** Outlet surface of a hollow channel. **B)** Inlet surface of a hollow channel. **C)** Bifurcation surface of a hollow channel. **D)** CAD model of the device with regions of interest highlighted. **E)** Stereo microscopic images of one bifurcation of the 3D printed, PDMS and Teflon^TM^ devices, corresponding to the region ‘C’ shown in the CAD model (D).


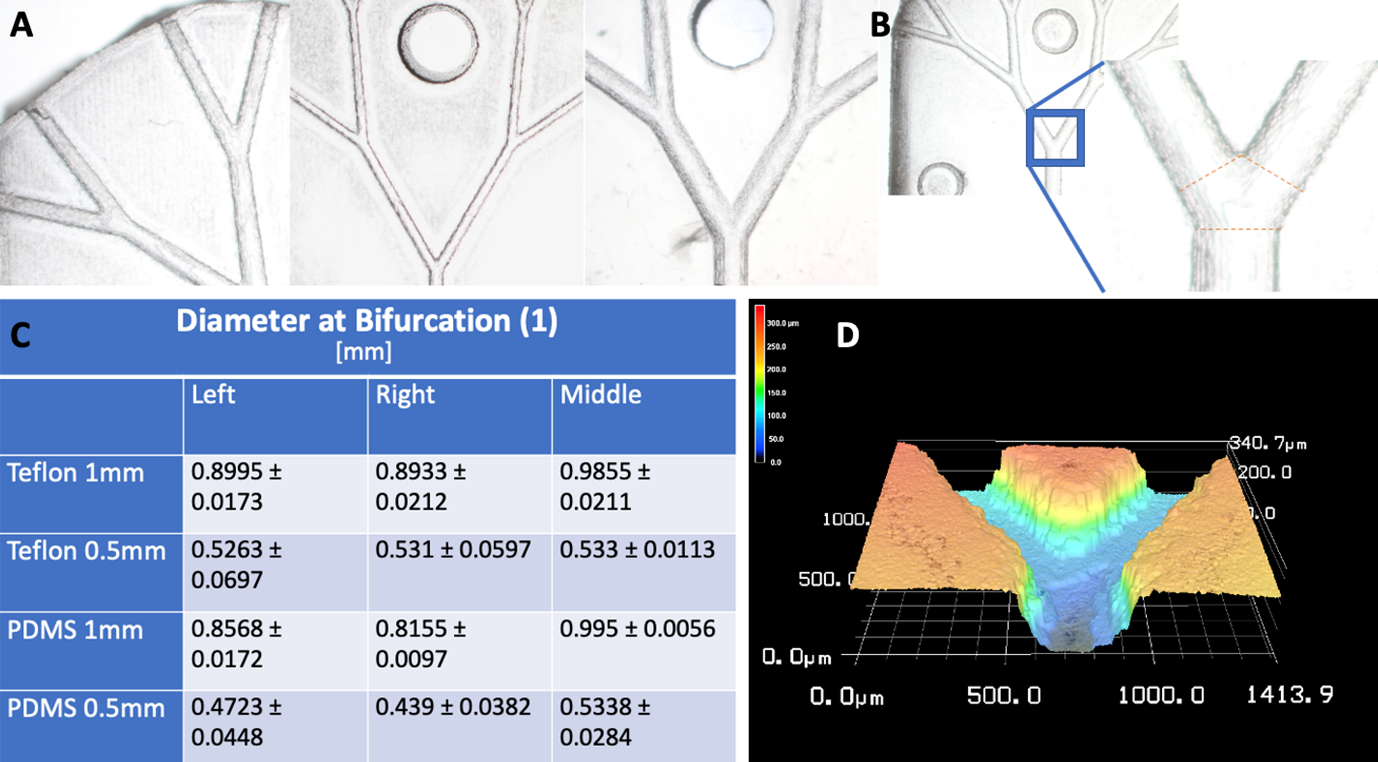


**Supplementary Figure S7:** **A)** From left to right: Stereoscope images of 1-mm, 0.5-mm PDMS and 1-mm Teflon^TM^ chip, **B)** Stereoscope image of 1-mm Teflon^TM^ device with the area of measurement highlighted (dashed lines), **C)** Table with mean and standard deviations, **D)** Profilometer image of 0.5-mm PDMS device**.**

**
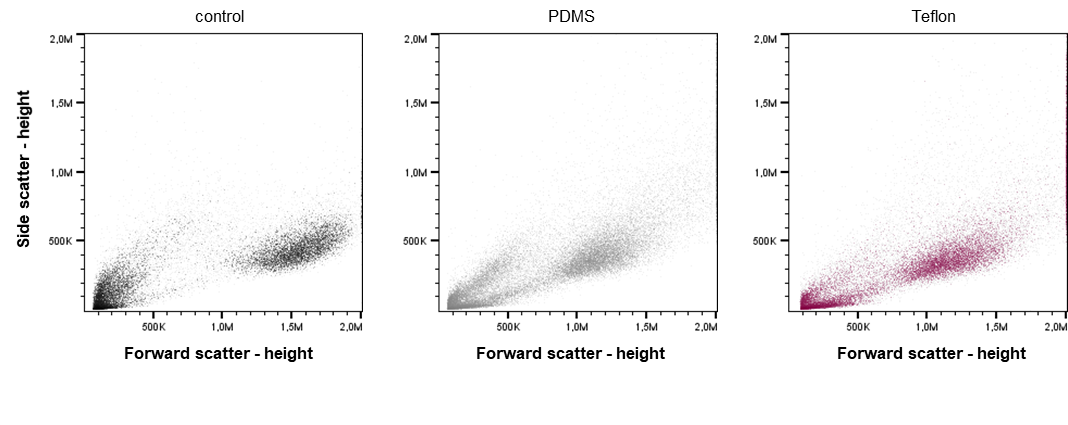
**

**Supplementary Figure S8:** Flow cytometry results in terms of forward vs. side scatter height for the control (left), PDMS- (middle) and Teflon^TM^ -cultured (right) cells**.**

## Supplementary Tables

**Supplementary Table S1:** Different mesh sizes for the tree-structured microvasculature model.

| **Mesh** | **Min size (µm)** | **Max size (µm)** | **Cells** | **Nodes** | **Edges** | **Faces** |
| --- | --- | --- | --- | --- | --- | --- |
| **Mesh 1** | 0.001 | 0.5 | 410017 | 1429925 | 33056 | 2061061 |
| **Mesh 2** | 0.01 | 0.5 | 223995 | 799796 | 69065 | 1094330 |
| **Mesh 3** | 0.08 | 1 | 98278 | 368702 | 69895 | 458525 |
| **Mesh 4** | 0.1 | 1 | 80422 | 259280 | 22641 | 371056 |

**Supplementary Table S2:** Different mesh sizes for the tree-structured microvasculature model.

| **Mesh** | **maximum**  **velocity** | | **AWA**  **wall shear stress** | | **AWA**  **pressure** | | **iterations** |
| --- | --- | --- | --- | --- | --- | --- | --- |
|  | value (mm/s) | % diff | value (Pa) | % diff | value (Pa) | % diff | count |
| **Mesh 1** | 13.7 | 0 | 0.27 | 0 | 9.14 | 0 | 144 |
| **Mesh 2** | 13.7 | 0.06 | 0.26 | -0.27 | 9.11 | -0.33 | 105 |
| **Mesh 3** | 23.8 | 42.68 | 0.26 | -0.37 | 9.45 | 3.59 | 76 |
| **Mesh 4** | 14.8 | -60.56 | 0.27 | 2.53 | 10 | 5.48 | 82 |

**Supplementary Table S3:** Mann-Whitney U-test results of the channel widths (left L, middle M, and right R) of the Teflon^TM^ (T) and PDMS (P) chips.

|  | **T_L_** | **P_L_** | **T_M_** | **P_M_** | **T_R_** | **P_R_** |
| --- | --- | --- | --- | --- | --- | --- |
| **T_L_** | - |  |  |  |  |  |
| **P_L_** | 0.057 | - |  |  |  |  |
| **T_M_** | n.a. | n.a. | - |  |  |  |
| **P_M_** | n.a. | n.a. | 0.343 | - |  |  |
| **T_R_** | 0.886 | n.a. | n.a. | n.a. | - |  |
| **P_R_** | n.a. | 0.029 | n.a. | n.a. | 0.029 | - |

# References

1. Ren K, Chen Y, Wu H. New materials for microfluidics in biology. Current Opinion in Biotechnology. 2014 Feb 1;25:78–85.

2. Ren K, Dai W, Zhou J, Su J, Wu H. Whole-Teflon microfluidic chips. Proceedings of the National Academy of Sciences. 2011 May 17;108(20):8162–6.

3. M. Nightingale A, Hassan S ul, Makris K, T. Bhuiyan W, J. Harvey T, Niu X. Easily fabricated monolithic fluoropolymer chips for sensitive long-term absorbance measurement in droplet microfluidics. RSC Advances. 2020;10(51):30975–81.

4. Sosnowski M, Krzywanski J, Grabowska K, Gnatowska R. Polyhedral meshing in numerical analysis of conjugate heat transfer. EPJ Web Conf. 2018;180:02096.

5. Amatoury M, Merheb V, Langer J, Wang XM, Dale RC, Brilot F. High-throughput Flow Cytometry Cell-based Assay to Detect Antibodies to N-Methyl-D-aspartate Receptor or Dopamine-2 Receptor in Human Serum. J Vis Exp. 2013 Nov 23;(81):50935.
